# Polycystic Ovary Syndrome: A Literature Review With a Focus on Diagnosis, Pathophysiology, and Management

**DOI:** 10.7759/cureus.47408

**Published:** 2023-10-20

**Authors:** Shrutika V Waghmare, Amardeep Shanoo

**Affiliations:** 1 Department of Obstetrics and Gynecology, Jawaharlal Nehru Medical College, Datta Meghe Institute of Higher Education and Research, Wardha, IND

**Keywords:** pcos treatment, insulin resistance, hyperandrogenism, diagnostic criteria, polycystic ovary syndrome

## Abstract

In females with polycystic ovarian syndrome (PCOS), the most prevalent endocrine condition is chronic anovulation and hyperandrogenism. This illness influences females from conception to death, posing several risks to the health of a female, thus reducing the quality of life. It also increases the rates of mortality and morbidity. The first years of puberty are when PCOS symptoms first show. Menstrual irregularities, anovulation, and acne are features of both PCOS and typical puberty in females. There are many various phenotypes that fall under the same illness, so it is necessary to examine each one independently because they may need different treatments and result in different outcomes. Depending on the diagnostic criteria, approximately 6%-20% of females in the reproductive age group are believed to be affected by PCOS. As long as PCOS is still a syndrome, no single diagnostic indicator, such as hyperandrogenism or polycystic ovary (PCO), can be used to make a clinical diagnosis. The management of females with PCOS depends on the symptoms. These could include menstruation problems, androgen-related symptoms, or infertility caused by ovulatory disruption. In females with PCOS, anovulation is linked to low follicle-stimulating hormone (FSH) levels and a halt in antral follicle growth during the last stages of maturation. The condition may be treated surgically with laparoscopic ovarian drilling or medically with medications such as aromatase inhibitors, metformin, glucocorticoids, clomiphene citrate (CC), tamoxifen, or gonadotropins. Patients will experience different androgenic symptoms, such as hirsutism, acne, and/or baldness. Patients who appear with these troubling symptoms need to receive appropriate care. The review emphasizes the role it plays in the management of various conditions.

## Introduction and background

**Figure 1:**
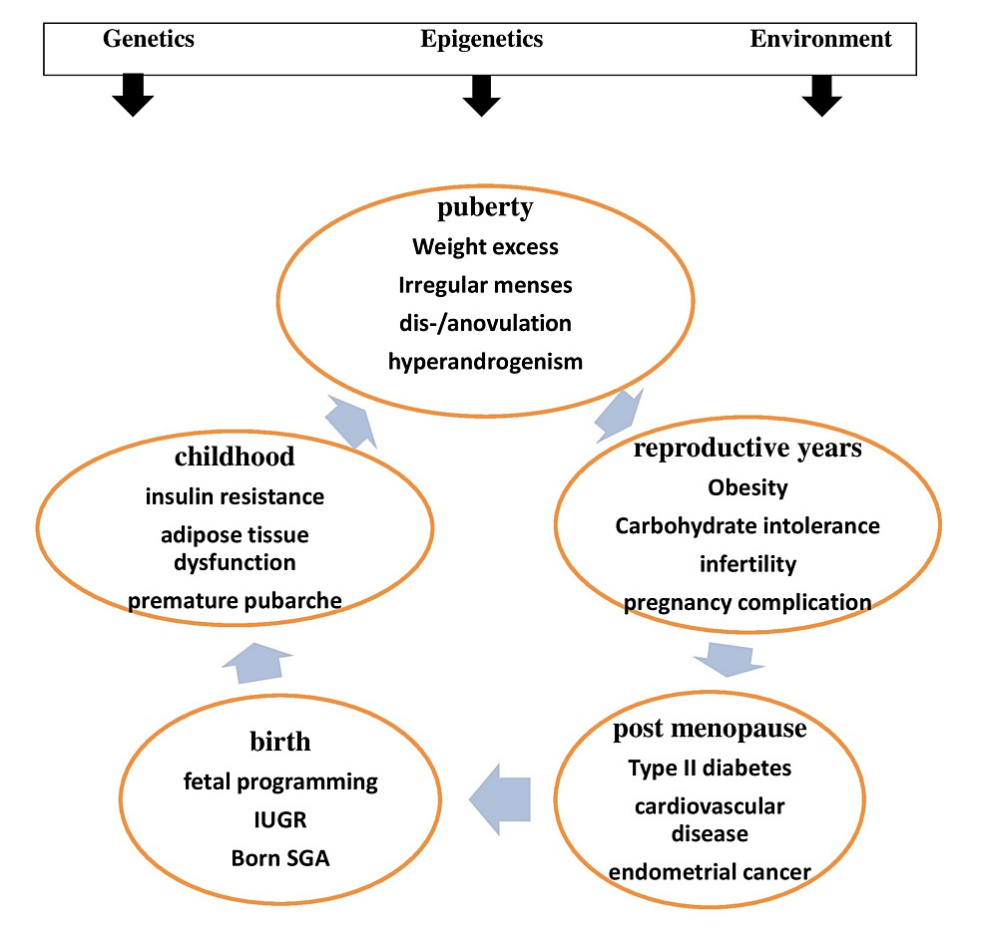
Clinical manifestations of PCOS in different stages of life of females Credits: Shrutika Waghmare IUGR, intrauterine growth retardation; SGA, small for gestational age; PCOS, polycystic ovarian syndrome

Worldwide, affecting many females in the reproductive age group, polycystic ovary syndrome (PCOS) is considered to be a heterogeneous endocrine disorder characterized by hyperandrogenism, ovulation dysfunction, and the morphology of polycystic ovary (PCO) [1]. Depending on the parameters defined, PCOS has a reported prevalence in the community of 6%-10% [2]. PCOS affects females from the moment of birth to the moment of death, providing a range of health hazards that may reduce their quality of life. It also contributes significantly to morbidity and mortality in the reproductive age group. With the most recent medical literature, the aim of this review is to summarize the medical effects from the start of reproductive life to its end (Figure 1) [3].

**Figure 2:**
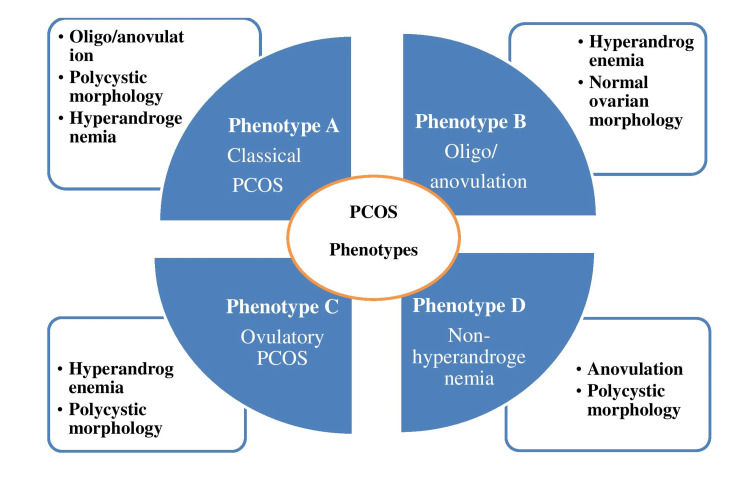
Four different phenotypes of polycystic ovarian syndrome (PCOS) Credits: Shrutika Waghmare

Sixteen phenotypes with various metabolic and reproductive effects may exist depending on the characteristics of the disease taken into consideration. There were four main phenotypes reported based on the Rotterdam criteria (Figure 2) [2,3]. The 2003 Rotterdam Consensus Workshop determined that PCOS is a syndrome of ovarian dysfunction on the basis of the cardinal features of hyperandrogenism and the morphology of polycystic ovary (PCO) [4]. The data suggests that this disorder affects between 6% and 8% of females globally if diagnosed using the National Institutes of Health 1990 criteria. As a result, it can be regarded as one of the most common disorders overall and also the most common endocrine abnormality in the reproductive age group [4].

## Review

Diagnostic criteria

Females with polycystic ovarian syndrome have three different sets of diagnostic criteria. Each set includes various combinations of polycystic ovarian morphologic characteristics, hyperandrogenism, and ovulatory failure (Table 1) [5,6].

**Table 1:** Revised diagnostic criteria of PCOS Diagnostic criteria modified from Doc Ina Ob Gyne [5] and McCartney and Marshall [6] PCOS, polycystic ovarian syndrome; AEPCOS, Androgen Excess and PCOS Society; NIH, National Institutes of Health; ESHRE-ASRM, European Society of Human Reproduction and Embryology-American Society for Reproductive Medicine

Criteria	ESHRE-ASRM 2003 "Rotterdam criteria"	AEPCOS 2006	NIH Workshop 2012
Hyperandrogenism	Any two of the following three features: menstrual irregularity, hyperandrogenism, and polycystic ovaries on ultrasound	Hyperandrogenism	Hyperandrogenism
Ovulation dysfunction	Any two of the following three features: menstrual irregularity, hyperandrogenism, and polycystic ovaries on ultrasound	Either ovulatory dysfunction or polycystic ovaries on ultrasound required	Ovulatory dysfunction required
Polycystic ovarian morphologic features	Any two of the following three features: menstrual irregularity, hyperandrogenism, and polycystic ovaries on ultrasound	Either ovulatory dysfunction or polycystic ovaries on ultrasound required	Not applicable

All needed the exclusion of additional underlying hormonal problems or malignancies, which include non-classic adrenal hyperplasia, thyroid disease, androgenic tumors, Cushing's syndrome, and hyperprolactinemia [5].

It is estimated that 60%-80% of PCOS females have high circulating androgen levels. Clinically, hyperandrogenism can present with excessive hair growth also known as hirsutism, seborrhea, and acne. Another feature is male pattern baldness also known as androgenic alopecia [4,5]. Hyperandrogenemia must be assessed with accurate androgen tests. When total testosterone is at lower levels in females, it may not be reliable. Hence, mass spectrometry-based total testosterone is preferred. In females with PCOS, free testosterone is the most sensitive test for hyperandrogenemia [6].

Ovulatory dysfunction is often characterized by irregular menstrual cycles that occur at fewer than 21-day interval or greater than 35-day interval. The most prevalent cause of oligo/amenorrhea in premenopausal females is polycystic ovarian syndrome (PCOS), and the majority of females with PCOS are insulin-resistant [7]. Obesity, hyperinsulinemia, and a more central fat distribution are all risk factors for irregular menstruation and type 2 diabetes [8]. The mechanism of insulin action on the ovaries causes hyperinsulinemia, which causes excessive androgen synthesis and peripheral conversion of androgens to estrogens in adipose tissue, resulting in menstrual cycle disruptions and lower conception rates. Higher estrogen levels cause menstrual irregularities and anovulation via negative feedback at the hypothalamic-pituitary level [9].

Morphologically, an ovary is considered to be polycystic if 12 or more than 12 antral follicles of 2-9 mm in diameter are present in each ovary with an ovarian volume of more than 10 mL [4,10]. Many females in this antral follicle count are asymptomatic diagnosed via transvaginal transducers with frequencies of 8 MHz or higher. Many experts use the criteria of 25 antral follicles instead of 12 for greater specificity. A skilled ultrasonographer is required for the proper use of any criterion; a report of "polycystic ovaries" without further qualification is insufficient for diagnostic purposes [10].

Pathophysiology

An important aspect of PCOS is androgen excess, found in 60%-80% of those who have the condition. High androgen production has the side effects of hirsutism and hyperandrogenism. In fact, the most prevalent abnormality observed in PCOS is hyperandrogenism, which significantly contributes to the aberrant hormones that cause PCOS pathogenesis. Elevated levels in the blood of free testosterone are a common symptom of hyperandrogenism [11].

Primary Ovarian Pathophysiology

Typically, only one follicle undergoes sequential terminal maturation and ovulation due to interactions influencing follicular growth. At delivery, there are only about 2-3 million primordial ovarian follicles, down from a high of about 6-7 million during mid-gestation. Controlling the pace at which new primordial follicles are added to the expanding pool is crucial for preserving fertility and the ovarian reserve since these follicles are then constantly pulled from this pool [12]. Follicles that are active and dormant coexist in a dynamic equilibrium. Anti-Müllerian hormone (AMH), follicle-stimulating hormone (FSH), and androgens are out of balance in PCOS, which causes follicular arrest [13]. Theca cells produce androgens when luteinizing hormone (LH) levels are high, but when FSH levels are low and androgens cannot be converted to estradiol, no dominant follicle may be chosen, leading to prolonged anovulation [14]. This equilibrium is tightly regulated by the hormone AMH, which is released by granulosa cells and prevents primordial follicles from maturing into primary follicles. In light of this, PCOS is characterized by an increase in the size of the small follicles, followed by a halt in growth that causes a unique polycystic form. Some ideas contend that follicles in an ovary with PCOS differ significantly from those in a normal ovary [15].

Insulin Resistance (IR)/Hyperinsulinemia

Females with PCOS frequently display insulin resistance (IR) and hyperinsulinemia, regardless of their level of adiposity or androgen levels [16]. Females with PCOS are at an increased risk of developing type 2 diabetes and impaired glucose tolerance [17]. Importantly, PCOS females experience tissue-selective IR. The adrenal gland and ovary are sensitive to insulin leading to steroid synthesis. However, the adipose tissue, skeletal muscle, and liver become more resilient to insulin's effects on metabolic activities. Because of this, certain tissues in females having PCOS show IR, while steroid-producing tissues still respond to insulin [18]. There may be additional contributing factors to IR and hyperinsulinemia, such as the pubertal rise in testosterone production. The association of hypoandrogenic state and IR has been previously studied because of the correlation between rare autoimmune disorders affecting the insulin receptors and hypoandrogenic features [19]. Insulin signaling is hampered by the buildup of ceramides and diacylglycerol (DAG) in the liver and muscle. By preventing the translocation of protein kinase B (Akt), a crucial modulator of insulin sensitivity, to the plasma membrane, intracellular ceramides can also negatively affect insulin signaling [20]. It is important to note that the disturbed regulation of insulin in the central nervous system has been connected to obesity and poor ovarian follicular maturation, which points toward more links between obesity, PCOS, and hyperinsulinemia in animals [21].

Neuroendocrine Alterations

Gonadotropin secretion changes in PCOS: The gonadotropins LH and FSH, which regulate ovulation, follicular dynamics, and the production of ovarian steroid, are found and released abnormally in PCOS, albeit they are not required for diagnosis. Since hyperandrogenism and ovulatory failure are the main characteristics of PCOS, it is plausible that changed gonadotropin secretion profiles may have an effect on these characteristics. In fact, elevated LH pulse frequency and/or amplitude, increased LH/FSH ratios, elevated LH levels in the blood, and relatively low FSH levels have all been observed in females with PCOS [22,23]. However, certain females have PCOS with hyperandrogenism, especially individuals who also have obesity, and have a non-elevated baseline or triggered LH levels, which also exhibit the diversity of the syndrome's manifestations (and etiology). Although the separation between gonadotropin-releasing hormone (GnRH) and LH has been observed in several models, it may help to explain why certain obese females with PCOS release less LH than others, despite the fact that LH is supposed to be the marker of GnRH pulses [24].

Other Metabolic and Endocrine Factors of GnRH Secretion in PCOS

AMH plays a previously unrecognized role in the positive regulation of GnRH-secreting neurons, according to recent research. A dose-dependent increase in the pulsatile production of LH has been seen in female mice after centrally administering AMH. The anti-Müllerian hormone receptor type 2 (AMHR2) receptors for AMH are found on neurons secreting GnRH and are stimulated more due to GnRH-dependent action. In this situation, the unregulated levels of AMH in PCOS may be a factor in LH hypersecretion. Although AMH might play a central role in PCOS-associated endocrine dysfunction, the neurosecretion of GnRH due to the effect of AMH is only demonstrated in control animals and not in PCOS models or patients [25]. Although increased GnRH/LH secretion is primarily caused by hyperandrogenism and perhaps other ovarian causes, raised insulin levels and IR may also play a role in these neuroendocrine changes [26]. In line with the secretory patterns of PCOS-afflicted females, insulin infusion in control females increased the frequency of LH pulses. In fact, it has been demonstrated that PCOS patients who are slim exhibit elevated basal LH levels leading to an increase in LH/FSH ratio. Another research including PCOS-afflicted females found that insulin treatment had no effect on LH pulsatility [26,27].

**Figure 3:**
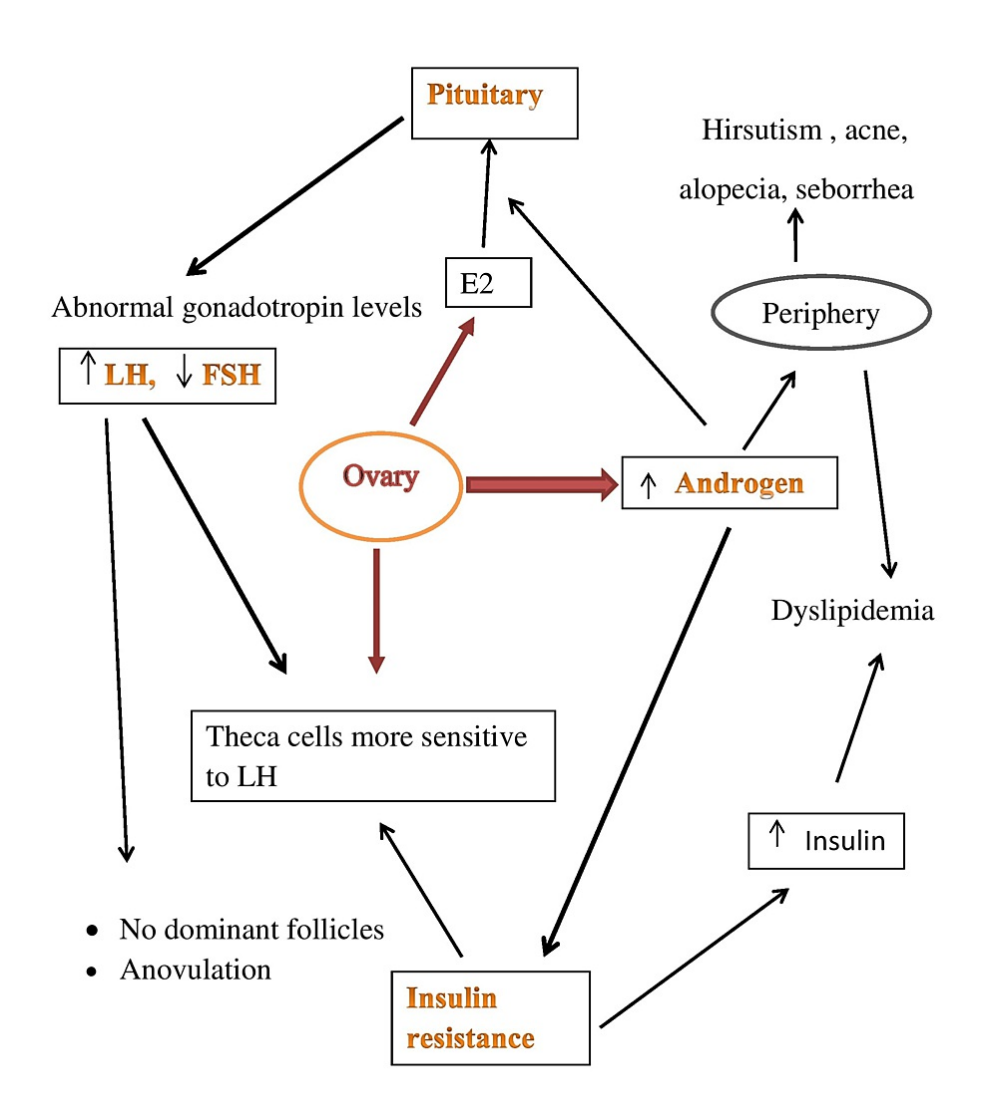
Summarized scheme regarding the pathophysiology of PCOS Credits: Shrutika Waghmare ↑, increased; ↓, decreased; LH, luteinizing hormone; FSH, follicle-stimulating hormone; E2, estradiol; PCOS, polycystic ovarian syndrome

A summary of the most representative molecular mechanisms of PCOS pathogenesis is presented in Figure 3 [5,11,22].

Pharmacological interventions

The key elements of PCOS treatment include symptom diagnosis and management. Infertility-causing anovulation, androgen-related symptoms, and irregular menstruation are a few instances of these. All the pharmaceutical and non-pharmacological therapies for PCOS are included in Table 2 [5,28,29].

**Table 2:** Commonly prescribed medications in PCOS FSH, follicle-stimulating hormone; LH, luteinizing hormone; ↑, increased; ↓, decreased; GnRH, gonadotropin-releasing hormone; HMG-CoA, 3-hydroxy-3-methylglutaryl coenzyme A; PCOS, polycystic ovarian syndrome

Drug	Mechanism of action	Purpose of therapy	Side effects
Estrogen and progestin			
Levonorgestrel/ethinyl estradiol	The normal pattern of the gonadotropin production of FSH and LH from the anterior pituitary gland is altered when ovulation is inhibited by negative feedback on the hypothalamus	Menstrual cyclicity, acne, and hirsutism	Breast tenderness, nausea and vomiting, weight gain, and acne
Desogestrel/ethinyl estradiol		Menstrual cyclicity	Depression, headache, skin rash, mood changes, urticaria, decreased or increased libido, and breast hypertrophy and tenderness
Cyproterone acetate		Menstrual cyclicity	Dysmenorrhea, nervousness, chloasma, dizziness, and edema
Drospirenone		Menstrual cyclicity, acne, and hirsutism	Menstrual irregularities, fatigue, mood changes, increased weight, and irritability
Progestins			
Medroxyprogesterone acetate	The inhibition of the secretion of pituitary gonadotropins and the prevention of follicular maturation and ovulation	Menstrual cyclicity	Amenorrhea, hot flash, weight gain or weight loss, abdominal pain, nervousness, and headache
Biguanides			
Metformin	↓Hepatic glucose production, ↑insulin sensitivity, and ↓intestinal absorption	Hyperinsulinemia, androgen excess, and anovulation	Nausea and vomiting (common), diarrhea, lactic acidosis, and metallic taste
Antiandrogens			
Spironolactone	Aldosterone receptor antagonists causes potassium retention and sodium and water excretion	Hirsutism and acne	Gynecomastia, hyperkalemia, and hypotension
Antiestrogens			
Clomiphene citrate	Increasing the pulsatile GnRH production from the hypothalamus and the release of pituitary gonadotropin by suppressing the normal estrogen negative feedback	Ovulation induction	Mood swings, ovarian enlargement, bloating, blurred vision, intermenstrual spotting, and menorrhagia
Aromatase inhibitors			
Letrozole	Nonsteroidal competitive aromatase enzyme inhibitor that catalyzes testosterone to estrogen conversion	Ovulation induction	Hot flashes, dizziness, arthralgia, headache, hypercholesterolemia, sweating increased, and bone pain
Simvastatin	↓HMG-CoA and ↓cholesterol biosynthesis	Hyperandrogenism and dyslipidemia	Worse insulin resistance, upper respiratory infections, nausea, and constipation
Atorvastatin			Nasopharyngitis, pain in extremity, and diarrhea

Oral Contraceptives

Oral contraceptive pills (OCPs) can be used to treat females who have no desire to conceive. These actions promote direct antagonistic feedback on LH secretion, which reduces ovarian androgen synthesis and hyperandrogenism. The insulin resistance that typically accompanies hyperandrogenism is not alleviated by the combined oral contraceptive pill. OCPs have been shown to worsen insulin resistance. The OCPs also tend to increase the likelihood of developing coagulatory and inflammatory problems in females both with and without PCOS. The OCPs used in PCOS are crucial because the majority of progestins have androgenic effects. The androgenic effect of progestin is a significant factor for the progestin component [4,30].

Antiandrogens

Cyproterone acetate (CPA), flutamide, and spironolactone are examples of antiandrogens that function by competitively blocking androgen-binding receptors or by lowering androgen production. Since antiandrogens reduce hirsutism and other androgen-related issues, they are generally used to treat PCOS. Although the antiandrogens function a little bit differently, they all inhibit the effects of testosterone. Antiandrogenic drugs such as spironolactone can cause irregular menstruation and, if the patient falls pregnant, can feminize a male fetus by competitively attaching to the androgen receptor. Therefore, spironolactone is mainly used in conjunction with OCPs to treat symptoms associated with PCOS [31].

Insulin Sensitizers

It has also been demonstrated that metformin and other insulin sensitizers, such as thiazolidinediones (TZD), can promote ovulation by reducing insulin resistance. The utilization of metformin is associated with improved ovulation, lower blood testosterone levels, and higher menstrual cyclicity. The biguanide metformin works by lowering intestinal glucose absorption, enhancing insulin sensitivity in peripheral tissues, and suppressing hepatic glucose synthesis. Thiazolidinediones causes fluid retention leading to weight gain; however, metformin can lead to weight loss [31,32].

Clomiphene Citrate (CC)

Clomiphene citrate (CC) is the first-choice treatment for PCOS-affected females to trigger ovulation. It is widely used to treat anovulation due to its demonstrated efficacy in inducing ovulation. Obese patients often need high doses of CC; however, these high doses may increase the likelihood of multiple pregnancies. CC is started at 50 mg/day on day (D) 2 or 3 of the menstrual cycle and increased to 250 mg/day over five days [33]. Although CC treatment increases ovulation rates by 60%-85%, it only marginally raises pregnancy rates by 30%-40%, with a higher chance of multiple pregnancies (5%-7%) [34].

Aromatase Inhibitors

Aromatase inhibitors are a recently identified and promising class of ovulation-inducing medications. The conversion of testosterone into estradiol and estrone is prevented by selective aromatase inhibitors such as anastrozole and letrozole. They increase FSH production, lower estrogenic activity, and relieve the hypothalamus of unwanted feedback [35]. The usual dosage of letrozole for PCOS patients is 2.5-7.5 mg within D3 to D7 of the menstrual cycle or 20 mg all at once on day 3 of the cycle. In a meta-analysis study that contrasted aromatase inhibitors with clomiphene citrate, there were statistically significant results in favor of aromatase inhibitors as it has a higher number of pregnancies and live births [36]. According to a recent systematic review and meta-analysis of studies on ovulation induction, letrozole is superior to clomiphene citrate in terms of the greater number of ovulation rate, pregnancy, and live birth for WHO Group 2 (including PCOS) anovulatory females, as well as for pregnancy rate in therapy [37].

## Conclusions

PCOS is a complicated condition involving several organ systems that starts during the early pubertal age. As the number of factors linked to the pathophysiology grows, more and more data points to hyperandrogenism as a crucial factor influencing a variety of tissues. It is believed that a number of factors, including obesity, inflammation, inactivity, and epigenetic alterations, may exacerbate this condition; however, the role of these factors in the pathogenesis of PCOS is not fully understood. The normal course of treatment for this ailment is symptomatic therapy utilizing a number of drugs, such as methods of oral contraceptive pills, oral antidiabetics, or antiandrogens, as there is currently no established or specialized pharmaceutical treatment for it. To better understand the pathophysiology and, thus, target the mechanism with the appropriate drug, there is still much to discover and investigate.
